# Lung ultrasound features predict admission to the neonatal intensive care unit in infants with transient neonatal tachypnoea or respiratory distress syndrome born by caesarean section

**DOI:** 10.1007/s00431-020-03789-z

**Published:** 2020-09-19

**Authors:** Antonio Poerio, Silvia Galletti, Michelangelo Baldazzi, Silvia Martini, Alessandra Rollo, Sofia Spinedi, Francesco Raimondi, Maurizio Zompatori, Luigi Corvaglia, Arianna Aceti

**Affiliations:** 1 Pediatric Radiology Unit, AOU Bologna, Bologna, Italy; 2 Neonatal Intensive Care Unit, AOU Bologna, Department of Medical and Surgical Sciences, University of Bologna, Bologna, Italy; 3 Department of Translational Medical Sciences, Division of Neonatology, University “Federico II”, Naples, Italy

**Keywords:** Lung ultrasound, Newborn, Caesarean section, Neonatal intensive care unit

## Abstract

We aimed to evaluate the reliability of lung ultrasound (LU) to predict admission to the neonatal intensive care unit (NICU) for transient neonatal tachypnoea or respiratory distress syndrome in infants born by caesarean section (CS). A prospective, observational, single-centre study was performed in the delivery room and NICU of Sant’Orsola-Malpighi Hospital in Bologna, Italy. Term and late-preterm infants born by CS were included. LU was performed at 30’ and 4 h after birth. LU appearance was graded according to a previously validated three-point scoring system (3P-LUS: type-1, white lung; type-2, black/white lung; type-3, normal lung). Full LUS was also calculated. One hundred infants were enrolled, and seven were admitted to the NICU. The 5 infants with bilateral type-1 lung at birth were all admitted to the NICU. Infants with type-2 and/or type-3 lung were unlikely to be admitted to the NICU. Mean full-LUS was 17 in infants admitted to the NICU, and 8 in infants not admitted. In two separate binary logistic regression models, both the 3P- and the full LUS proved to be independently associated with NICU admission (OR [95% CI] 0.001 [0.000–0.058], *P* = .001, and 2.890 [1.472–5.672], *P* = .002, respectively). The ROC analysis for the 3P-LUS yielded an AUC of 0.942 (95%CI, 0.876–0.979; *P*<.001), while ROC analysis for the full LUS yielded an AUC of 0.978 (95%CI, 0.926–0.997; *P*<.001). The AUCs for the two LU scores were not significantly different (*p* = .261).

*Conclusion*: the 3P-LUS performed 30 min after birth proved to be a reliable tool to identify, among term and late preterm infants born to CS, those who will require NICU admission for transient neonatal tachypnoea or respiratory distress syndrome.

**Table:** 

**What is known** • *Lung ultrasound (LU) has become an attractive diagnostic tool in neonatal settings, and guidelines on point-of-care LU in the neonatal intensive care unit (NICU) have been recently issued.* • *LU is currently used for diagnosing several neonatal respiratory morbidities and has been also proposed for predicting further intervention, such as NICU admission, need for surfactant treatment or mechanical ventilation in preterm infants.*	
**What is new** • *LU performed 30′ after birth and evaluated through a simple three-point scoring system represents a reliable tool to identify, among term and late preterm infants born to caesarean section, those with transient neonatal tachypnoea or respiratory distress syndrome who will require NICU admission.* • *LU performed in the neonatal period confirms its potential role in ameliorating routine neonatal clinical management.*

**Electronic supplementary material:**

The online version of this article (10.1007/s00431-020-03789-z) contains supplementary material, which is available to authorized users.

## Introduction

Birth by caesarean section (CS) has been linked to an increased risk of several neonatal respiratory morbidities, including respiratory distress syndrome (RDS) and transient tachypnoea of the newborn (TTN). Recent data also suggest that the negative effects of CS on respiratory function might extend beyond the neonatal period, leading to respiratory morbidities in the long term, such as obstructive sleep apnoea [1] and asthma [2]. Active labour before delivery enhances lung liquid clearance soon after birth [3]. Consistently, a recent study performed using lung ultrasound (LU) has shown that healthy term infants delivered by spontaneous vaginal delivery (VD) or in-labour CS had a more rapid clearance of lung fluids compared with those delivered by elective CS; regardless of the mode of delivery, however, all the infants achieved a normal lung appearance at LU within 20 min from birth [4].

Given its concordance with conventional x-ray [5], its validation against a wide number of imaging techniques [6] and the excellent inter-observer agreement [7], LU has become an attractive diagnostic tool in neonatal settings, and guidelines on point-of-care LU in the neonatal intensive care unit (NICU) have been recently issued [8]. LU is currently used for diagnosing several neonatal respiratory morbidities and has been also proposed for predicting further intervention, such as NICU admission [9], need for surfactant treatment or mechanical ventilation in preterm infants [10, 11]. However, despite LU has been acknowledged by recent international evidence-based recommendations as a useful diagnostic tool for neonatal respiratory morbidities [8, 12], and specific algorithms for diagnosing the most common respiratory diseases have been proposed [13], the routine adoption of point-of-care LU in the NICU is still experiencing some limitations [7, 14]. The implementation of routine LU in the NICU would allow to further optimise the management of newborns with, or at risk of, respiratory morbidities.

In the present study, we aimed to investigate the reliability of LU, performed at birth, in predicting NICU admission due to TTN or RDS in term and late preterm infants delivered by CS.

## Materials and methods

This prospective, observational, single-centre study was performed at the Delivery Room and NICU of Sant’Orsola-Malpighi Hospital in Bologna, Italy, from December 2016 to March 2017. Term and late preterm infants (gestational age [GA] ≥ 34 weeks) born by CS, either planned or in-labour, were included. Infants with an antenatal diagnosis of congenital heart disease, other major congenital malformation or chromosome abnormality were excluded. Written informed consent was obtained from the parents/legal guardians of the recruited infants.

A clinical evaluation of the infants enrolled was performed in the delivery room within 30 min after birth. Admission to the NICU was based on the occurrence, in the delivery room, of clinical signs of respiratory distress which did not resolve within 30 to 60 min of life. Respiratory distress was defined by the evidence of a respiratory rate > 60 breaths/min, associated with at least one of the following: tachycardia (heart rate higher than 160 bpm), intercostal or supraclavicular retractions, nasal flaring, expiratory grunting and hypoxia (pre-ductal oxygen saturation in room air lower than 85%) [15]. Upon admission, infants were classified according to the physio-pathology of the respiratory distress as having TTN or RDS. TTN, which is caused by a delayed fluid absorption in the lungs after birth, presents with signs of mild respiratory distress and is usually self-limiting; however, a small proportion of infants with TTN can require NICU admission due to the need of oxygen supplementation [16]. RDS, or hyaline membrane disease, is caused by insufficient surfactant production, thus being more common among preterm infants, who often require exogenous surfactant replacement [15].

Prenatal and neonatal clinical variables which could influence the risk for NICU admission were collected, including maternal morbidities during pregnancy such as gestational diabetes, hypertension and hypothyroidism, singleton vs. twin pregnancy, administration of antenatal steroids, intrauterine growth restriction, gender, mode of delivery (planned vs. in-labour CS), gestational age (GA), birth weight (BW), Apgar score at 5′, arterial cord blood pH and base excess.

For each infant, LU examination was performed in the delivery room at approximately 30′ from birth (T0) and then repeated at 4 h of life (T1), with infants lying in a supine position. Each LU was performed by the same operator in order to rule out potential inter-observer variability using a portable sonographic scanner (Cx50, Philips Healthcare, Eindhoven, The Netherlands) equipped with a high-frequency (7.5–13 MHz) linear hockey stick probe with a depth of 3 cm, with the focus position located at the pleural line. Transversal (probe parallel to the ribs) and longitudinal (probe perpendicular to the ribs) scans of the anterior (hemiclavear line) and lateral chest (middle axillary line) wall were recorded; posterior areas were not examined in order to minimise infants’ handling. Pleural sliding was observed to rule out pneumothorax [13].

LU images were stored and reviewed offline by a second operator, who was blinded to the infants’ clinical status, using the three-point lung ultrasound scoring system (3P-LUS) validated by Raimondi et al. [9, 17]. According to this scoring system, a type 1 lung is characterised by a hyper echoic appearance caused by coalescent B lines (the so-called white lung—Supplementary Fig. 1a), a type 2 lung by numerous non-compact B lines (black and white lung—Supplementary Fig. 1b), and a type 3 lung is the normal aerated lung, with the dominance of A lines (black lung—Supplementary Fig. 1c). In order to analyse in deeper detail the reliability of this three-image score, the full LUS proposed by Brat and colleagues was also calculated [18]. Meconium aspiration syndrome, pneumonia and any type of neonatal acute RDS [19] were excluded a posteriori.

The study protocol was approved by the “Area Vasta Emilia Centro” Independent Ethical Committee (CE-AVEC; study ID 60/2017/O/Sper) and was conducted in compliance with the Declaration of Helsinki.

### Statistical analysis

The target for sample size calculation was the number of NICU admissions for TTN or RDS in a given period in our NICU among term and late preterm infants born by CS. Specifically, the sample size was calculated as follows: the number of CS performed in 2015 in our hospital was approximately 950, and, among infants born by CS, the percentage of those with GA ≥ 34 weeks who were admitted to the NICU due to respiratory distress was approximately 7.5%. Thus, given a confidence interval of 95% and a margin of error of 5%, we calculated that we would have needed to include at least 96 infants in our study to be representative of the population.

Statistical analyses were performed using IBM SPSS Statistics v 20.0.0 (Armonk, NYC, USA), except for comparative ROC analysis, which was performed using the online free version of MedCalc. A *P* value ≤ 0.05 was considered as statistically significant.

Data distribution was evaluated using the Kolmogorov-Smirnov test and continuous variables were then expressed as mean (standard deviation [SD]) or median (interquartile range [IQR]) as appropriate. The study population was divided into two groups according to NICU admission. Clinical, laboratory and ultrasonographic characteristics were compared between the two groups using independent sample *t* test or chi-square test as appropriate. A single-step binary logistic regression model (enter method) was built to evaluate the association of clinical and ultrasonographic characteristics on the study outcome (NICU admission). The goodness-of-fit (Hosmer-Lemeshow test) and the between-variable collinearity were also evaluated.

To evaluate the relationship between a LU showing a bilateral type 1 lung appearance and NICU admission, sensitivity, specificity, positive and negative predictive value of the 3P-LUS were evaluated. Positive and negative likelihood ratios (LR) were also calculated.

Furthermore, given the possibility of inhomogeneous lung appearance, a further calculation was performed by considering as true-positive infants those with at least one type 1 lung: in this respect, true-positive (TP) infants were defined as those with at least one type 1 lung admitted to the NICU, true-negative (TN) as those with types 2–3 lung who remained in the neonatal nursery, false-positive (FP) as those with at least one type 1 lung who remained in the nursery, false-negative (FN) as those with types 2–3 lung who were admitted to the NICU.

For both calculations, the specificity of the LUS was defined as TN/(TN + FP), sensitivity as TP/(TP + FN); positive predictive value (PPV) as TP/(TP + FP), negative predictive value (NPV) as TN/(TN + FN). Values were expressed with 95% confidence intervals (CI).

Receiver operating characteristic (ROC) analysis was used to evaluate the ability of both the 3P-LUS and the full LUS score to predict NICU admission: areas under the curves (AUCs) and cut-off values showing the highest sensitivity were reported. The AUCs were compared using the method proposed by De Long et al. [20].

## Results

One hundred infants (51 male) were studied. The study flowchart is reported in Fig. S2 (supplementary materials). Mean gestational age was 37 weeks + 5 days (SD 3 weeks + 5 days) and mean birth weight was 2965 g (SD 504 g). All the infants were delivered by CS (86 planned, 14 in-labour). None of the mothers had chorioamnionitis, pre-eclampsia or pre-existing hypertension, while sixteen mothers had gestational diabetes and nineteen had hypothyroidism. The eight infants who received antenatal steroids were all late preterm.

At 30′ after birth, 84 infants had a homogeneous appearance in the two lungs: 5 infants were assigned a type 1 score in both lungs, 62 a type 2 score and 17 a type 3 score. The remaining 16 infants had inhomogeneous lung appearance: 6 had a type 1 and type 2 lung, and 10 had a type 2 and type 3 lung.

Seven infants were admitted to the NICU: 5 developed clinical signs of respiratory distress in the delivery room, while 2 were admitted due to low birth weight and started to show signs of respiratory distress upon admission. All of them received nasal CPAP for 24–48 h: six were diagnosed as having TTN, and one was classified as having RDS and was given surfactant through the InSurE procedure (intubation-surfactant-extubation). The length of NICU stay ranged between 5 and 26 days (median 9 days).

Table 1 depicts clinical variables associated with NICU admission: infants who were admitted to the NICU had significantly lower GA, lower BW and were more likely to be late preterm and to show clinical signs of respiratory distress at 30′ min after birth. No differences in terms of gender, mode of delivery (elective vs. in-labour CS), Silverman score, 5-min Apgar score or cord blood pH were documented between infants admitted to the NICU and those who were not.

**Table 1:** Clinical characteristics associated to NICU admission. Variables are reported as mean (standard deviation) or number (percentage) as appropriate. A *P* value < 0.05 is considered as statistically significant

	Overall population (*n* = 100)	Infants admitted to the NICU (*n* = 7)	Infants not admitted to the NICU (*n* = 93)	*P* value
Late preterm	13 (13%)	5 (71%)	8 (9%)	< 0.001
Silverman score	0.97 (1.98)	3.14 (2.79)	0.81 (1.62)	0.069
5-min Apgar score	9.71 (0.66)	9.57 (0.63)	9.72 (0.67)	0.57
Gender (male)	51 (51%)	6 (85.7%)	45 (48.4%)	0.112
In-labour C-section	14 (14%)	1 (14%)	13 (14%)	1
Maternal hypertension/pre-eclampsia	5 (5%)	0 (0)	5 (5.4%)	1
Maternal diabetes	16 (16%)	0 (0)	16 (17%)	0.594
Maternal hypothyroidism	19 (19%)	3 (43%)	16 (17%)	0.127
Antenatal steroids administration (full course)	8 (8%)	3 (43%)	5 (5.4%)	0.010
Cord blood arterial pH	7.29 (0.1)	7.33 (0.1)	7.29 (0.1)	0.484
Signs of respiratory distress at 30′	22 (22%)	5 (71%)	17 (18%)	0.005

LUS for each study group and the results of between-group comparisons are detailed in Table 2. Significantly lower 3P-LUS (*P* = 0.005) and higher full LUS (*P* < 0.001) were observed in infants admitted to the NICU compared with those who were not admitted. None of the infants showed any LU evidence of pneumothorax.

**Table 2:** Lung ultrasound characteristics associated to NICU admission. Variables are reported as raw number (percentages) for the three-image ultrasound score (LUS) and as mean (standard deviation) for the full LUS. A *P* value < 0.05 is considered as statistically significant

	Overall population (*n* = 100)	Infants admitted to the NICU (*n* = 7)	Infants not admitted to the NICU (*n* = 93)	*P* value
Three-image LUS				0.005
Both type 1 lungs	5 (5%)	5 (71.4%)	0	
One type 1 lung, one type 2 lung	6 (6%)	1 (14.3%)	5 (5.4%)	
Both type 2 lungs	62 (62%)	1 (14.3%)	61 (65.6%)	
One type 2 lung, one type 3 lung	10 (10%)	0	10 (10.8%)	
Both type 3 lungs	17 (17%)	0	17 (18.3%)	
Full LUS	9 (5)	17 (2)	8 (4)	< 0.001

Two binary logistic regression models including the 3P-LUS or full LUS and signs of respiratory distress were built (see Table 3). Late preterm status and antenatal steroids were not included in the regression model as strictly overlapping with the occurrence of respiratory distress at 30 min. The regression model confirmed a significant association between NICU admission, 3P-LUS (*P* = 0.001) and full LUS (*P* = 0.002). The *P* values of the Hosmer and Lemeshow test were > 0.05 for both models.

**Table 3:** Results of binary logistic regression models

	*R*^2^	B	SE	OR (95% CI)	*P* value
Three-image LU score	0.307	−7.362	2.301	0.001 (0.000–0.058)	*0.001*
Respiratory distress at 30 min		2.484	1.644	11.987 (0.478–300.64)	0.131
Full LU score	0.324	1.061	0.344	2.890 (1.472–5.672)	*0.002*
Respiratory distress at 30 min		1.435	1.682	4.202 (0.156–113.47)	0.393

Dependent variable: NICU admission

Significant p-values are shown in italics

The reliability of the 3P-LUS to predict NICU admission was first calculated by considering as true-positive only infants with both type 1 lungs: this score had a specificity of 100% (95% CI 95.4–100%) and a sensitivity of 71.4% (95% CI 61.4–79.8%); PPV was 100% (95% CI 95.4–100%), and NPV 97.9% (95% CI 92.1–99.6%). Negative LR was 0.286 (95% CI 0.179–0.456); no calculation of positive LR was possible, as the number of FP infants was zero.

When analysing data to clarify the potential role of a single type 1 lung in predicting NICU admission, specificity was 94.6% (95% CI 87.7–97.9%), and sensitivity was 85.7% (95% CI 77.0–91.6%); PPV was 54.5% (95% CI 44.3–64.4%), and NPV was 98.9% (95% CI 93.6–99.9%). Negative LR was 0.15 (95% CI 0.11–0.22) and positive LR was 15.94 (95% CI 11.74–21.66).

Four hours after birth, none of the infants presented with an inhomogeneous white lung. Most infants (69%) showed a normal LUS, with at least one type 3 lung, while the remaining 31% had a bilateral type 2 lung. Back-sliding (LUS worsening over time [4]) was seen in only one infant who was not admitted to the NICU and scored 3 at birth and 2 at 4 h of life on both lungs.

**Fig. 1:**
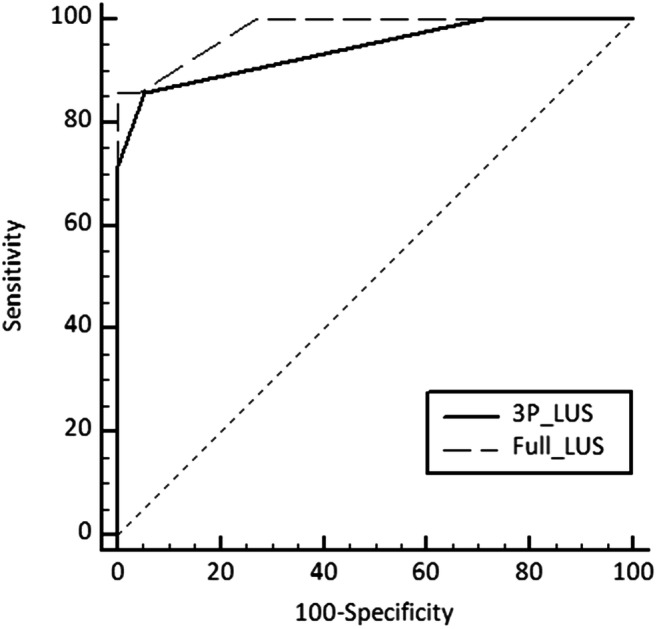
Comparative ROC analysis, including both the three-image (3P-LUS) and the full lung ultrasound score (full-LUS), performed using the method by DeLong et al. [20]

The ROC analysis for the 3P-LUS yielded an AUC of 0.942 (95%CI, 0.876–0.979; *P* < 0.001); the ROC analysis for the full LUS yielded an AUC of 0.978 (95%CI, 0.926–0.997; *P* < 0.001). The AUCs for the two LU scores were not significantly different (difference between areas 0.036; standard error 0.045 [95%CI − 0.027 to 0.099], *P* = 0.261; Fig. 1).

## Discussion

The present study confirms the reliability of early LU to predict NICU admission in term and late preterm infants. Furthermore, our data show that, in infants born by CS, either elective or in-labour, lung features detected by LU and described using an easy-to-perform three-point LUS can distinguish between infants who will require further intervention and those who will likely need only routine care.

When both lungs show an ultrasound type 1 pattern, the positive predictive value for NICU admission is excellent (100%). This is in line with previous findings by Raimondi et al., who evaluated the reliability of LU performed between 1 and 2 h after birth in late preterm and term infants born both by CS and VD in predicting NICU admission [9]. In addition, by examining the LU pattern of each lung separately, our data show that, when the white lung pattern affects a single lung, the positive predictive value is much lower (54.5%). Furthermore, when both lungs show a type 2 and/or type 3 pattern, admission to the NICU is very rare, as documented by the very high calculated negative predictive value (approximately 98% for both calculations).

This latter observation could be useful for the routine management of infants born by CS, especially in clinical settings where no neonatal intensive care is available on site. We might speculate that a 2 to 3 LUS in both lungs in the delivery room could prompt the return of infants born by CS to their mothers, limiting the interruption of skin-to-skin contact and maternal-infant bonding which is often seen after caesarean delivery. On the other side, our data suggest that infants with at least one type 1 lung at birth should be monitored carefully as, in the presence of a single white lung, NICU admission is unlikely but not to be excluded with certainty. It must be acknowledged, however, that the low number of infants with bilateral type 1 lung included in the present study limits the generalisability of the study results, warranting further multicentre trials aimed at confirming the current findings.

In the present study, no difference in LUS between infants delivered by in labour and planned C-section was documented at 30 min of life. A previous study performed by Martelius et al. compared lung liquid clearance in infants born via VD vs. CS, showing that, despite a significant decrease in the abundance of B-lines in both groups at 24 h after birth, liquid clearance was slower in infants born by CS [21]. Further studies compared LU findings among infants born via VD, in-labour CS and planned CS: although these studies documented a faster liquid clearance during the first minutes of life in infants born in the presence of labour [21], all the infants achieved a certain degree of liquid clearance within 20 min of life [4, 22]. Furthermore, complete airway liquid clearance was achieved within the first 4 h of life: similarly, none of the infants in the present study showed a LUS lower than 2 at the 4-h examination, and no back-sliding to a white lung pattern was documented over time.

A number of different LUS have been proposed for the evaluation of ultrasound lung appearance in the neonatal period: recently, an eighteen-point score, which combines ultrasound data from three areas for each lung, was validated by Brat et al. [18] and further adopted in a recent multicentre trial which evaluated infants with TTN [23]. The score, which allows a comprehensive evaluation of different lung areas, proved to correlate with oxygenation indexes and to be reliable in predicting the need of surfactant administration among preterm infants receiving non-invasive respiratory support [10]. The score validated by Raimondi et al., which was used in the present study, is a simpler three-point score which allows a global and rapid evaluation of each lung: this score was previously used to predict NICU admission [9] and non-invasive ventilation failure in preterm infants [17], and to describe lung appearance at birth until complete fluid clearance in term and preterm infants [24]. The results of the comparative ROC curve analysis performed in our study between the three-image LUS and the full score proposed by Brat and colleagues showed a good agreement between the two in predicting NICU admission for TTN or RDS in infants born by CS. To note, the full LUS has the advantage over the three-point score to be able to detect and measure the extension of lung areas with a C-pattern (consolidations, tissue-like areas, irregular areas with loss of aeration), and thus should be preferred and calculated in clinical situations when a consolidative process is suspected.

Some authors also used a modified, zero-to-three point version of this latter score in order to describe the specific lung appearance during the initiation of breathing, prior to the establishment of the pleural line [22]. An additional 5-step scale was proposed by Martelius et al. to describe the evolution of B-lines and the changes occurring in static lung compliance during the first 24 h of life [25]. When performing LU, the choice of the score to be used is probably dependent upon the specific clinical or research setting: a simple three-point score such as the one used in the present study might be the right choice for neonatal units where no intensive care is available and where the possibility to rely on a diagnostic tool which is easy to use and has high inter-observer agreement might help clinicians to make prompt clinical decisions. However, it has to be acknowledged that the choice of this scoring system has some limitations, as it provides a simplified description of lung appearance and is not reliable in non-homogeneous lung disorders such as pneumonia, meconium aspiration, sepsis and lung haemorrhage.

At present, no specific training curriculum is available for neonatal LU, and this constitutes a barrier to a more widespread use of LU in everyday clinical practice in the NICU [26]. A recent study by Gomond-Le Goff et al. documented a good inter-rater agreement and reliability in neonatal LU interpretation, irrespective of the probe and rater expertise. Furthermore, the authors provided useful information for implementing LU in neonatal clinical practice by documenting the existence of an “expertise-probe” interaction factor, according to which the use of non-linear probes by novice operators is associated with the lowest agreement and reliability of LU [27]. Recently, a simplified image system similar to the one used in the present study was introduced after a short formal training in a low-income setting: the simplified scoring system, although less accurate than the full LUS, proved to be useful to recognise RDS and TTN, providing a non-invasive and easily available tool to be integrated to clinical evaluation [28].

Beyond TTN and RDS [29], LUS in neonatal medicine has proved its usefulness in the detection of several other conditions, such as pneumothorax, pleural effusions, consolidations and atelectasis [7], and will hopefully give new insights in the management of non-respiratory diseases such as congenital heart disease [30]. In addition, in a nearby future, the field on neonatal LU will likely benefit from technological advances in image acquisition and analysis [31]. Some limitations to a widespread use of LU in the NICU still exist, including the risk of inter-observer disagreement in settings where the training level is low [32]. Future studies should aim at confirming the reliability of LUS as a diagnostic tool in the delivery room and NICU, also overcoming present limitations to its routine clinical application.

The results of the present study strengthen the reliability of LU, performed in the first minutes of life and evaluated through a simple scoring system, to predict accurately the need for NICU admission in term and late preterm infants, giving new insight into the potential of this technique to ameliorate routine neonatal clinical management.

## Electronic supplementary material

Supplementary Figure 1 Ultrasound appearance of neonatal lung according to the scoring system validated by Raimondi et al. [9, 17]. Type 1 lung (white lung) (a): uniform hyper echoic appearance caused by the presence of coalescent B-lines with irregular thickening of the pleural line; type 2 lung (black and white lung) (b): numerous non-compact B-lines; type 3 lung (black, normal lung) (c): absence of B-lines and presence of A-lines, horizontal hyper echoic lines parallel to the pleural line. For each figure, (A) shows longitudinal scans and (B) transverse scan (PNG 5360 kb)

High resolution image (TIF 578 kb)

Supplementary Figure 2 Flow chart depicting patients screening and enrolment (PNG 5360 kb)

High resolution image (TIF 86 kb)

## Abbreviations

3P-LUS: 3-point lung ultrasound score
BW: Birth weight
CS: Caesarean section
FN: False negative
FP: False positive
GA: Gestational age
IQR: Interquartile range
LU: Lung ultrasound
LUS: Lung ultrasound score
NICU: Neonatal intensive care unit
NPV: Negative predictive value
PPV: Positive predictive value
RDS: Respiratory distress syndrome
SD: Standard deviation
TN: True negative
TP: True positive
TTN: Transient tachypnoea of the newborn
