# Supplementary figures and images for: Lung ultrasound features predict admission to the neonatal intensive care unit in infants with transient neonatal tachypnoea or respiratory distress syndrome born by caesarean section
Source: Eur J Pediatr. 2020 Sep 19;180(3):869–76. doi: 10.1007/s00431-020-03789-z (PMC7886822; doi:10.1007/s00431-020-03789-z)

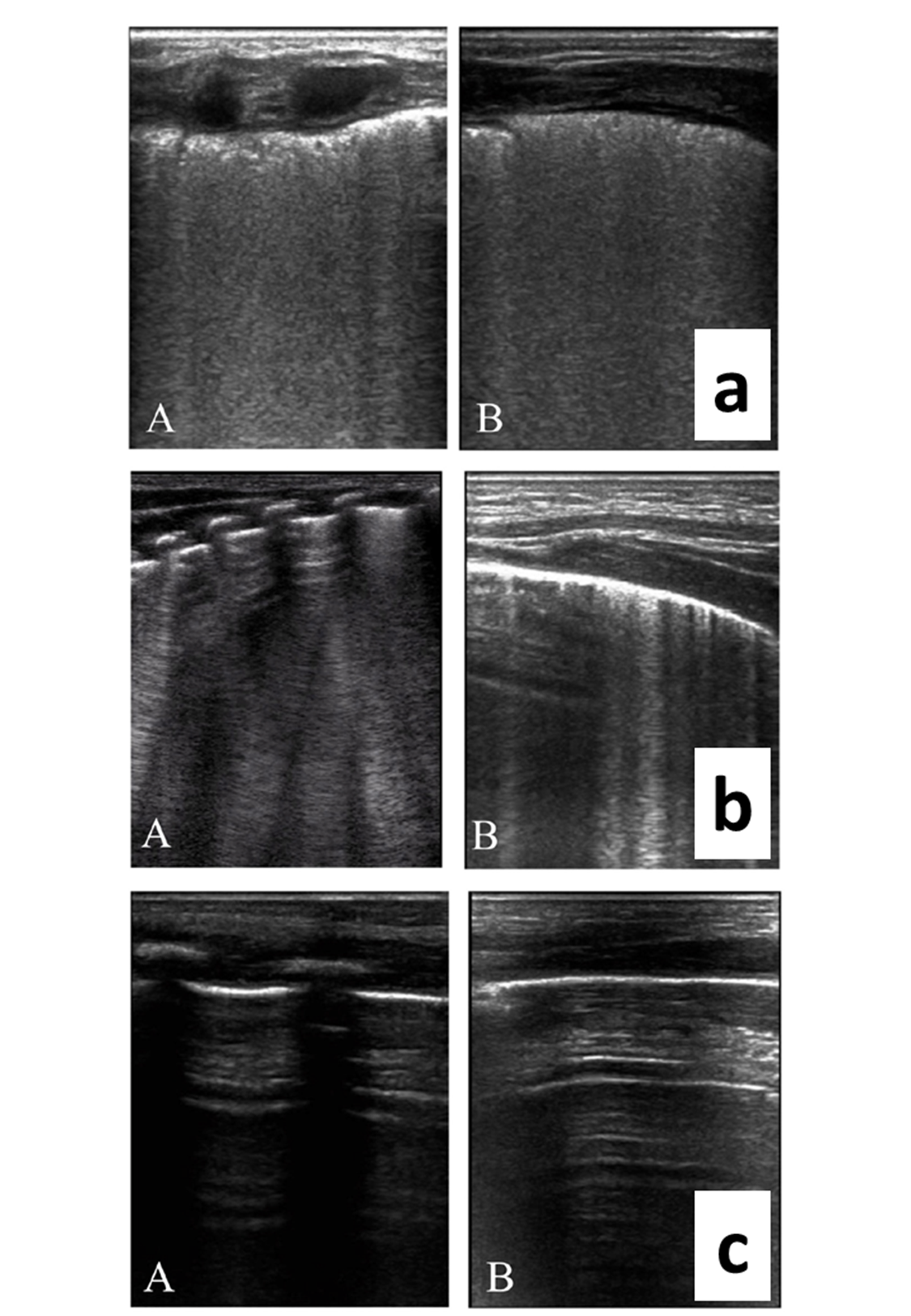

Supplement: Supplementary file 1 — Ultrasound appearance of neonatal lung according to the scoring system validated by Raimondi et al. [9, 17]. Type 1 lung (white lung) (a): uniform hyper echoic appearance caused by the presence of coalescent B-lines with irregular thickening of the pleural line; type 2 lung (black and white lung) (b): numerous non-compact B-lines; type 3 lung (black, normal lung) (c): absence of B-lines and presence of A-lines, horizontal hyper echoic lines parallel to the pleural line. For each figure, (A) shows longitudinal scans and (B) transverse scan (PNG 5360 kb) [file 431_2020_3789_Fig2_ESM.png]

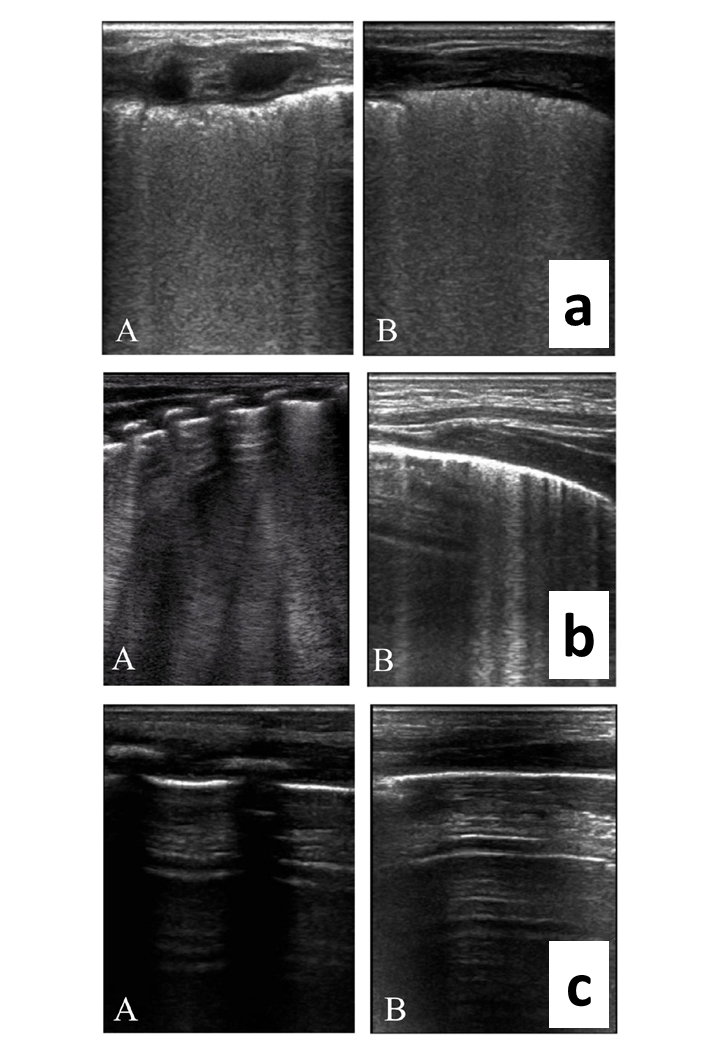

Supplement: Supplementary file 2 — High resolution image (TIF 578 kb) [file 431_2020_3789_MOESM1_ESM.tif]

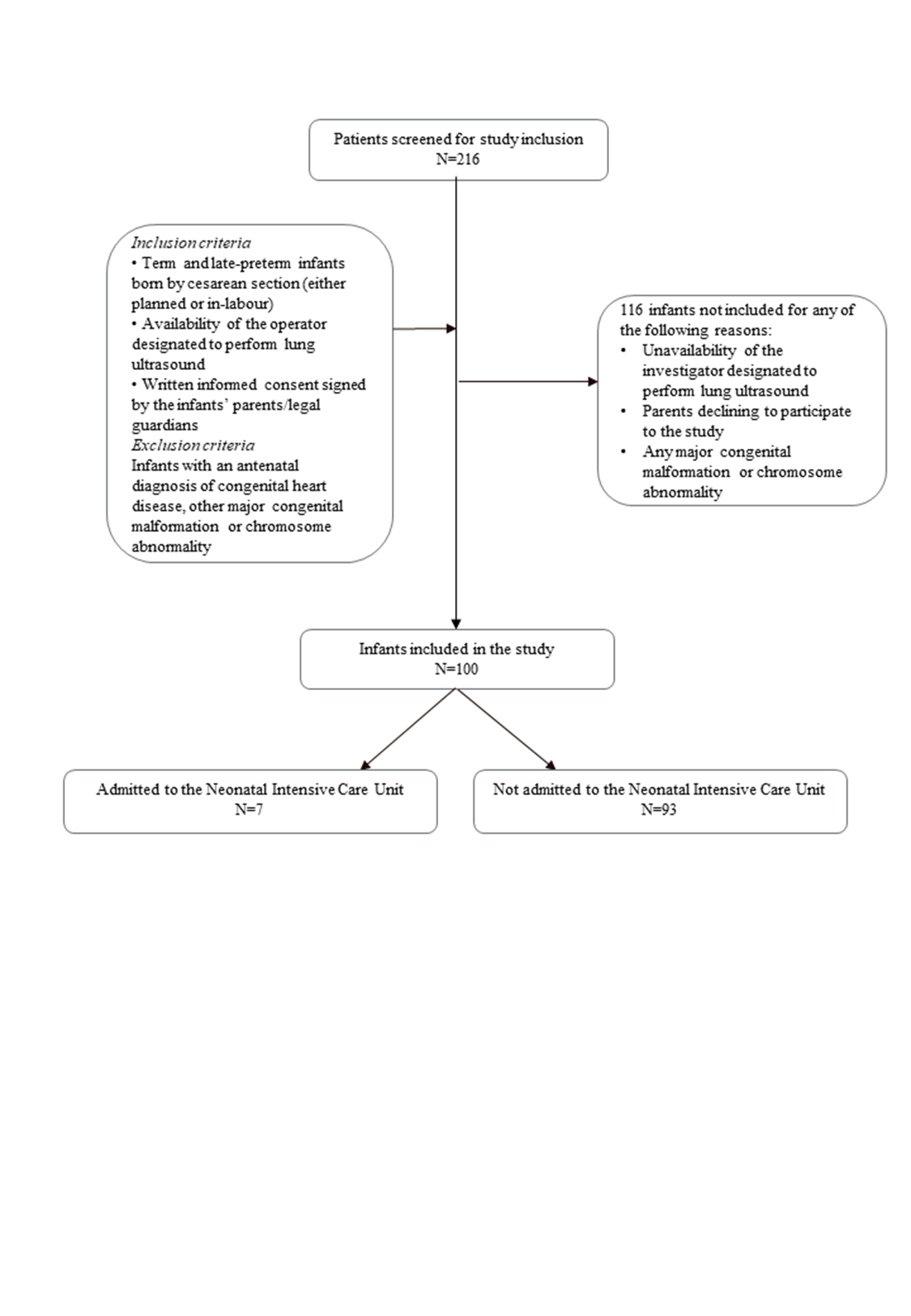

Supplement: Supplementary file 3 — Flow chart depicting patients screening and enrolment (PNG 5360 kb) [file 431_2020_3789_Fig3_ESM.png]

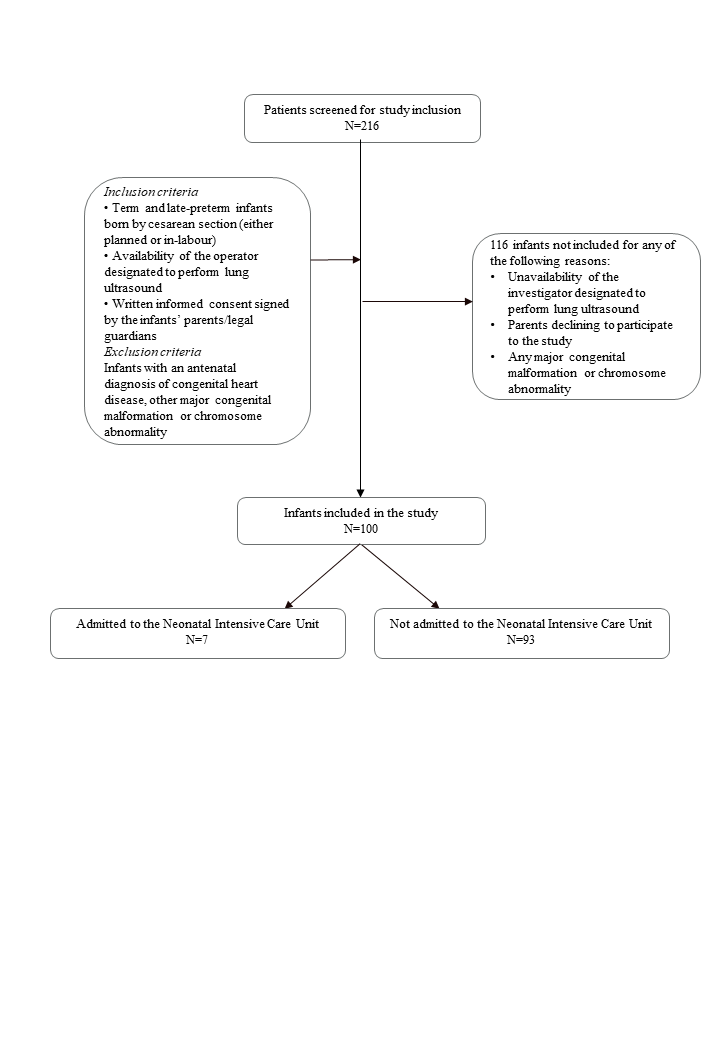

Supplement: Supplementary file 4 — High resolution image (TIF 86 kb) [file 431_2020_3789_MOESM2_ESM.tif]
